# Cyanobacterial Akinete Distribution, Viability, and Cyanotoxin Records in Sediment Archives From the Northern Baltic Sea

**DOI:** 10.3389/fmicb.2021.681881

**Published:** 2021-06-15

**Authors:** Steffaney M. Wood, Anke Kremp, Henna Savela, Sultana Akter, Vesa-Pekka Vartti, Saija Saarni, Sanna Suikkanen

**Affiliations:** ^1^Marine Research Centre, Finnish Environment Institute, Helsinki, Finland; ^2^Leibniz Institute for Baltic Sea Research Warnemünde, Rostock, Germany; ^3^Biotechnology, Department of Life Technologies, University of Turku, Turku, Finland; ^4^Radiation and Nuclear Safety Authority, Helsinki, Finland; ^5^Department of Geography and Geology, University of Turku, Turku, Finland

**Keywords:** cyanobacteria, resting stages, life cycles, microcystin, nodularin, resurrection, *ndaF*, *mcyB*

## Abstract

Cyanobacteria of the order Nostocales, including Baltic Sea bloom-forming taxa *Nodularia spumigena, Aphanizomenon flosaquae*, and *Dolichospermum* spp., produce resting stages, known as akinetes, under unfavorable conditions. These akinetes can persist in the sediment and germinate if favorable conditions return, simultaneously representing past blooms and possibly contributing to future bloom formation. The present study characterized cyanobacterial akinete survival, germination, and potential cyanotoxin production in brackish water sediment archives from coastal and open Gulf of Finland in order to understand recent bloom expansion, akinete persistence, and cyanobacteria life cycles in the northern Baltic Sea. Results showed that cyanobacterial akinetes can persist in and germinate from Northern Baltic Sea sediment up to >40 and >400 years old, at coastal and open-sea locations, respectively. Akinete abundance and viability decreased with age and depth of vertical sediment layers. The detection of potential microcystin and nodularin production from akinetes was minimal and restricted to the surface sediment layers. Phylogenetic analysis of culturable cyanobacteria from the coastal sediment core indicated that most strains likely belonged to the benthic genus *Anabaena*. Potentially planktonic species of *Dolichospermum* could only be revived from the near-surface layers of the sediment, corresponding to an estimated age of 1–3 years. Results of germination experiments supported the notion that akinetes do not play an equally significant role in the life cycles of all bloom-forming cyanobacteria in the Baltic Sea. Overall, there was minimal congruence between akinete abundance, cyanotoxin concentration, and the presence of cyanotoxin biosynthetic genes in either sediment core. Further research is recommended to accurately detect and quantify akinetes and cyanotoxin genes from brackish water sediment samples in order to further describe species-specific benthic archives of cyanobacteria.

## Introduction

Since the mid-twentieth century, anthropogenic eutrophication and climate variability have led to an increase of harmful cyanobacterial blooms in the Baltic Sea (Finni et al., 2001; Suikkanen et al., 2013; Funkey et al., 2014). Widespread cyanobacteria blooms now occur annually in the Baltic Sea and can negatively impact the local ecosystem, human health, and economy (Sivonen and Jones, 1999; Karjalainen et al., 2007; Burford et al., 2019). Despite the annual occurrence of cyanobacteria blooms, the life cycle of such bloom-forming cyanobacteria remains poorly understood (Hense and Beckmann, 2006; Suikkanen et al., 2010).

Cyanobacteria of the order Nostocales can produce resting stages, known as akinetes, under unfavorable conditions (Hori et al., 2003; Komárek, 2010). These akinetes persist in the sediment and germinate if favorable conditions return, simultaneously representing past blooms and contributing to the initiation of future blooms (Livingstone and Jaworski, 1980; Wood et al., 2009; Legrand et al., 2017b). Akinetes are characterized by their augmented size, thick cell walls, non-motility, and spheroid or oval shape (Kaplan-Levy et al., 2010; Sukenik et al., 2019). These characteristics lend toward the ability to withstand harsh environmental conditions. Therefore, akinetes often serve as an effective overwintering and recruitment strategy, similar to protist resting cysts, in temperate regions such as the Baltic Sea (Suikkanen et al., 2010). Many factors can influence akinete germination of different cyanobacteria species from benthic environments, including sediment resuspension, sufficient maturation time, light, temperature, oxygen, and nutrients (Huber, 1985; Adams and Duggan, 1999; Baker and Bellifemine, 2000; Hori et al., 2003; Kaplan-Levy et al., 2010; Myers et al., 2010).

Various techniques have been used to study sedimentary persistence, viability, and recruitment of cyanobacterial akinetes. Studies on cyanobacterial akinete sedimentation and records in the sediment have addressed akinete abundance, viability, and DNA from sediment samples in lakes (Livingstone and Jaworski, 1980; Wood et al., 2009; Legrand et al., 2016, 2017a,b, 2019) and brackish water environments (Suikkanen et al., 2010; Bormans et al., 2020). Via the incubation of sediment samples in culture media, germination experiments are commonly employed to assess the viability and recruitment conditions of akinetes (Livingstone and Jaworski, 1980; Wood et al., 2009; Suikkanen et al., 2010). Genetic methods have also been widely used to investigate cyanobacterial presence and potential cyanotoxin production in sediments by amplification of the 16S rRNA gene (e.g., Rinta-Kanto et al., 2009; Savichtcheva et al., 2011; Legrand et al., 2017a) and various cyanotoxin biosynthesis genes such as the microcystin synthetase encoding *mcyA*, *mcyB*, and *mcyD* (e.g., Rinta-Kanto et al., 2009; Savichtcheva et al., 2011; Monchamp et al., 2016; Legrand et al., 2017a, 2019; Pilon et al., 2019; Bormans et al., 2020) or *ndaF*, related to nodularin biosynthesis (e.g., Cegłowska et al., 2018). Recently, metabarcoding has been adopted to the selection of genetic tools used for the study of sediment cyanobacterial assemblages (e.g., Monchamp et al., 2016; Pilon et al., 2019; Yan et al., 2019). These studies have found akinetes to be viable up to more than a 1000 years after their sedimentation and identified the occurrence of microcystin (*mcyB*) and anatoxin (*anaC*) genes in 6,700 years old sediment layers (Legrand et al., 2019). Thus, sediments may represent archives of past blooms (Livingstone and Jaworski, 1980; Räsänen et al., 2006; Wood et al., 2009; Legrand et al., 2017b).

Plankton resting stages present in the sediment of aquatic environments create a reservoir of genetic diversity, possibly allowing for the adaptation to changing environmental conditions (Jones and Lennon, 2010; Kremp et al., 2016). Sediment archives of living resting stages and/or DNA are increasingly recognized as powerful tools for the study of climate change impacts on aquatic life (Ellegaard et al., 2020). In the Baltic Sea, the approach has been successfully used to trace the dynamics of dinoflagellates and their evolutionary responses to changing environmental conditions through the past century (Hinners et al., 2017; Kremp et al., 2018).

Baltic Sea cyanobacteria blooms are dominated by nostocalean, filamentous, and nitrogen-fixing cyanobacteria, represented by *Nodularia spumigena*, *Aphanizomenon flosaquae*, and *Dolichospermum* (ex. *Anabaena*) species. Baltic Sea strains of *N. spumigena* and certain *Dolichospermum* spp. produce the hepatotoxic nodularin and microcystins, respectively (Sivonen et al., 1989; Karlsson et al., 2005; Halinen et al., 2007). These cyanotoxins threaten the safety of drinking water and recreational water use due to their ability to damage liver cells in mammals, potentially causing death in acute doses (Sivonen and Jones, 1999). Understanding life cycle strategies, particularly akinete formation and recruitment, of these bloom-forming and potentially cyanotoxin-producing genera, provides insight into future bloom initiation and cyanotoxin outbreaks, which remains vital to enhancing bloom prediction and mitigation strategies (Suikkanen et al., 2010; Wasmund, 2017).

This study investigated cyanobacterial akinete distribution, viability, and potential for cyanotoxin production in >40 to >400-year-old sediment archives from the northern Baltic Sea in order to assess cyanobacteria bloom expansion, the extent of cyanotoxins in past blooms, and species-specific life cycle strategies. We hypothesized that akinete abundance and presence of cyanotoxins would correspond with degradation of akinetes over time and the expansion of cyanobacterial blooms in the northern Baltic Sea since the mid-twentieth century. Cyanotoxin gene examination and phylogenetic analysis of culturable cyanobacteria strains was used to further investigate species-specific life cycle strategies by associating germinated strains with known Baltic Sea strains. Furthermore, akinete survival and germination potential was studied, to better understand their function as long-term seed banks and species-specific life cycle strategies of cyanobacteria in the northern Baltic Sea. This project aimed to contribute to a greater understanding of cyanobacteria and akinete resilience, diversity, and viability in the fluctuating northern Baltic Sea environment.

## Materials and Methods

### Sediment Core Collection and Processing

**FIGURE 1:**
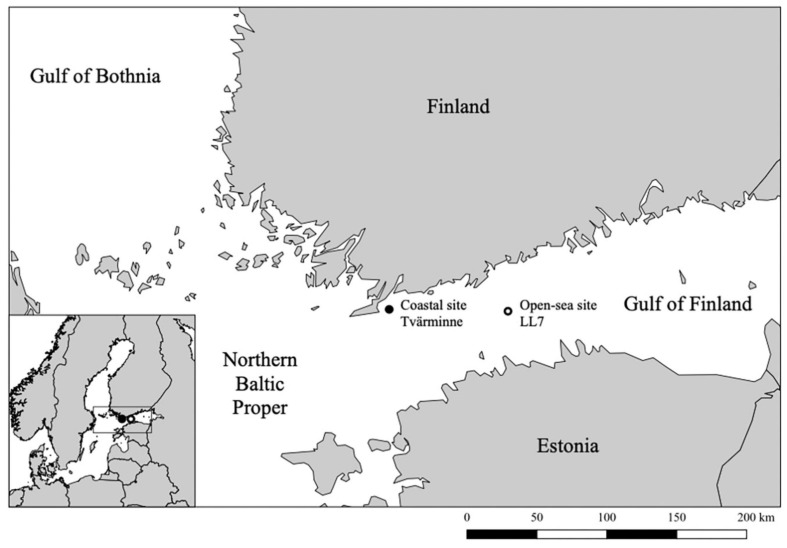
Map of the study area showing the location of the sediment core collection sites.

Sediment cores were obtained from the Gulf of Finland at a coastal site (35 m depth) near Tvärminne Zoological Station in July 2018 and at an open-sea site (approximately 100 m depth), LL7, in January 2019 (Figure 1) using a GEMAX gravity corer. Two replicate cores of approximately 40 cm length were taken at the coastal site, with one core reserved for sediment dating. A single core of 30 cm length (LL7-2019) was collected at the open-sea site. A replicate core was not taken from the open-sea site due to the availability of previous sediment dating data specific to the LL7 site (LL7-2015; Kremp et al., 2018). All cores were stored in their original tubes under cold and dark conditions until processing. Sediment layers were selected for analyses according to high dry to wet weight ratios, relative age, and for consistency across analyses.

Cores were extruded from sampling tubes using a piston and sliced into 1- to 2-cm layers. To minimize contamination, the outer 5 mm of each slice was removed using a small inner corer. Individual slices for dating were stored in plastic containers at 4°C and dark conditions. Individual slices for biological analysis were stored in plastic bags submerged in water to remove air at 4°C and dark conditions in order to ensure the preservation of akinetes (Kremp et al., 2018). Wet and dry weight of sediment slices was determined by placing aliquots (5 mL) of wet sediment in crucibles, weighing them and drying them in an UNE 400 Memmert Universal oven at 70°C for approximately 24 h, after which the mass of dried sediment samples was recorded.

### Sediment Dating

Wet sediment slices of one coastal core reserved for gamma-spectrometric dating were weighed and freeze-dried for 3 days in a Christ Beta 2–8 LD plus freeze dryer at 1.0 mbar and −60°C. Dry material was then reweighed. Dry sediment was analyzed using gamma-spectrometry at the Finnish Radiation and Nuclear Safety Authority (STUK) Laboratory in Helsinki, Finland. The relative age of each 2-cm sediment layer was determined using Cs-137 radionuclide analysis and certified National Physical Laboratory reference samples. Markers for Cs-137 dating were peaks corresponding to calendar years 1963 and 1986. These peaks were produced by nuclear weapon testing, accidents, and the Chernobyl disaster.

Dating of sediment core LL7-2015 from the open-sea site was conducted for previous research using Cs-137 and Pb-210 concentrations, constant initial concentration and constant rate of supply models (Kremp et al., 2018). Cores LL7-2015 and LL7-2019 were correlated based on clear physical sediment features such as variations in water content and wet-to-dry-weight ratio.

### Determination of Vertical Akinete Abundance

Akinetes present in the 20–63 μm fraction of wet sediment samples were quantified using light microscopy. Every 2- to 4-cm depth of sediment was counted from both cores to determine vertical akinete abundance. The 20–63 μm sediment fraction was selected because it likely contained the majority of cyanobacterial akinetes, which are on average one order of magnitude bigger than the average vegetative cell size (Adams and Duggan, 1999; Kaplan-Levy et al., 2010; de Tezanos Pinto et al., 2016). The 20–63 μm fraction also eliminated fine silt particles present in the 10–63 μm fraction. These particles were excluded to maximize light microscopy visibility and counting accuracy.

To obtain the 20–63 μm fraction, aliquots (2 mL) of sediment were diluted with 6 psu sterile-filtered seawater and sonicated on a constant duty cycle for 30 s at 20–40 kHz (Brandelin Sonoplus Sonicator HD 2200). The sonicated sediment slurry was sifted through 63 and 20 μm stainless steel mesh sieves with 6 psu sterile-filtered seawater. The remaining material on the 20 μm sieve was collected and stored in 15-mL polypropylene centrifuge tubes with 6 psu sterile-filtered seawater under dark conditions at 4°C. Akinetes from each triplicate sample were quantified using an inverted light microscope (Leica DMI 3000 B) with a 1-mL Gridded Sedgewick Rafter chamber (Wildlife Supply Company) at 200X magnification. Identification of akinetes followed Komárek (2013, 2016). A minimum of 400 cells (or all cells in the sample, if <400) were counted in each 1-mL sample. Recorded akinete counts and wet to dry weight sediment ratios were used to calculate the average abundance of akinetes present in dry sediment samples.

### DNA Extraction From Sediment

Genomic DNA (gDNA) was extracted from selected wet sediment samples from the coastal and open-sea core corresponding with sediment layers containing akinete abundance data. Up to 250 mg of wet sediment was transferred to PowerBead tubes (Qiagen) with a sterile, disposable Pasteur pipette. The mass of all samples was recorded. Tubes were bead-beat using a FastPrep-24 5G (MP Biomedicals) sample disruption instrument for two cycles of 6 m s^–1^ for 45 seconds with a 5 s break between cycles. DNA extraction was conducted using the DNeasy PowerLyzer Power Soil Kit (Qiagen) according to the manufacturer’s instructions, eluted with 100 μL molecular biology grade water, and stored at −20°C. Concentration and purity of extracted DNA was verified (NanoDrop 2000, Thermo Fisher Scientific).

### Cyanotoxin Extraction From Sediment and Detection by Non-competitive Immunoassay

Particulate cyanobacterial hepatotoxins microcystin and nodularin were measured from the 20–63 μm sediment fraction. Sediment core layers selected for hepatotoxin content analysis corresponded with those selected for determination of vertical akinete abundance. This included nine open-sea sediment core samples and 11 coastal sediment core samples.

From each selected sediment sample, 6 mL of wet sediment was sieved to obtain the 20–63 μm size fraction which was suspended in approximately 10 mL of sterile-filtered 6 psu seawater. This material was centrifuged (3,500 rpg for 10 min) and the supernatant was discarded. The resulting pellets were washed three times with 5 mL Milli-Q water to remove accumulated salts. Each pellet was suspended in 100 μL Milli-Q water, frozen at −20°C, and freeze-dried for approximately 12 h at 1.0 mbar and −60°C. Freeze-dried material was suspended in 1.2 mL 75% (v/v) methanol and sonicated in an ultrasonic bath for 15 min. The methanol extract was then transferred to a new vial for evaporation. The methanol extraction was repeated twice, and extracts were combined into one vial per sample. Methanol extracts were evaporated to dryness at room temperature under nitrogen flow for approximately 12 h. Samples were then reconstituted in 400 μL Milli-Q water and centrifuged to remove any remaining solids (3,500 rpg for 10 min). The supernatants were transferred into glass vials and stored at −20°C until used for detection of hepatotoxins by non-competitive immunoassay. Sample preparation procedure was based on methods previously described (Kankaanpää et al., 2001, 2009).

Non-competitive immunoassays were used to detect the presence of microcystins and/or nodularin in the sediment extracts. Each sample was initially analyzed with an assay that detects both microcystins and nodularin (Akter et al., 2016), with slight modifications. Using 25 μL well^–1^ of sample, the assay was performed in 75 μL well^–1^ reaction volume. The detection limit (expressed in microcystin-LR equivalents) of the immunoassay was 0.04 μg L^–1^, based on the average + 3 SD of the blank measurements. Similarly, each sample was also analyzed to detect any possible nodularin-R according to the method described by Akter et al. (2017) in reaction volume of 75 μL well^–1^ using 25 μL well^–1^ of sample. The detection limit of the nodularin-R immunoassay was 0.011 μg L^–1^. Finally, all hepatotoxin concentrations were converted to pg of hepatotoxin per g of dry weight. For more detailed description of the immunoassays, see Supplementary Data 1.

### Cyanobacterial Akinete Germination and Establishment of Clonal Cultures

Live cyanobacterial akinetes present in sediment layers were enumerated and quantified using the serial dilution culture-most probable number (SDC-MPN) method (Throndsen, 1978). Three and eight sediment layers were selected for analysis from varying depths within the coastal and open-sea cores, respectively. Aliquots (3 mL) of wet sediment were initially diluted 1:10 in 6 psu sterile-filtered seawater and sonicated on a constant cycle at 20–40 kHz for 30 s to separate akinetes from sediment particles. Sonication of the sediment slurry was completed on ice to approximately maintain the 4°C sediment storage conditions. The original 1:10 subsample was then serially diluted by 1:10 seven times, with 2 mL of the previous dilution and 18 mL sterile, nitrogen-free, 6 psu Z8xS media (Kotai, 1972). Only 1:10^3^ through 1:10^7^ dilutions were further incubated and each of these dilutions contained five replicates.

Apart from four open-sea core sediment layers, this procedure was completed in duplicate for all samples to undergo two temperature treatments, 4 and 16°C. These temperature conditions were used to simulate winter/early spring and summer conditions in the northern Baltic Sea. The open-sea core sediment layers 8–9, 10–11, 12–13, and 14–15 cm were only incubated in 16°C. Incubating samples were stored in 50-mL sterile tissue culture flasks under the selected temperature treatment and constant light conditions, approximately 100 μml photons m^–2^s^–1^.

Sediment slurry incubations were checked for cyanobacteria germination after approximately 4 and 8 weeks using an inverted light microscope with a 1-mL Sedgewick Rafter chamber at 200X magnification. The presence or absence of any cyanobacteria along with the specification of Nostocales taxa was recorded. Most probable number calculations were completed using the presence numbers recorded from the 1:10^3^ to 10^5^ dilution replicates and Throndsen (1978) Most Probable Number table. As the MPN calculations only required three dilutions, the 1:10^6^ and 1:10^7^ were excluded.

Identification of single filaments with morphological characteristics of *Dolichospermum* and *Nodularia* spp. followed Komárek (2013). Selected single filaments were isolated and cultured from selected SDC-MPN samples of the coastal sediment core containing these taxa. Approximately 1 mL of the germinated SDC-MPN sample was transferred to a 3-mL sedimentation chamber. The sample was examined for *Dolichospermum* and *Nodularia* spp. filaments using an inverted light microscope at 200X magnification. Single filaments of interest were selected using a sterile glass micropipette, washed three times with Milli-Q water, and transferred to a 24-well culture plate (Eppendorf) filled with 1:3 dilution of sterile, 6 psu Z8xS media and sterile-filtered 6 psu seawater. Well plates were then incubated at 16°C under constant light conditions, approximately 100 μmol photons m^–2^ s^–1^.

### DNA Extraction, 16S rRNA Gene Amplification and Phylogenetic Analysis of Clonal Cultures

Ten successful clonal cultures were selected for further analysis. Cells were collected approximately 48 h after the cultures were inoculated with fresh 6 psu Z8xS culture medium. A 10 mL subsample of each selected culture was concentrated (<100 mg), pelleted and then ground. gDNA was extracted using a Thermo Scientific GeneJET Plant Genomic DNA Purification Mini Kit according to the manufacturer’s instructions and stored at −20°C. DNA concentration and quality was determined spectrophotometrically (NanoDrop 2000).

PCR amplification of cyanobacterial 16S rRNA gene was conducted in 20 μL reactions containing 2 μL template (gDNA or molecular biology grade water for negative control), 1X Phusion HF Buffer, 0.4 U Phusion DNA Polymerase, 0.2 mM dNTP mix (all reagents Thermo Scientific), 0.4 μM 16S27F and 23S30R primers (Table 1; all primers manufactured by Integrated DNA Technologies). Thermal cycling (C1000 Thermal Cycler, Bio-Rad) was carried out as follows: 98°C, 30 s followed by 30 cycles of 98°C, 5 s; 66°C, 30 s; 72°C, 30 s followed by final 72°C for 5 min. Amplification success was verified using gel electrophoresis (Invitrogen E-Gel, General Purpose Agarose Gel, 1.2% w/v agarose; Invitrogen E-Gel Power Snap Gel Electrophoresis Device). PCR products were purified using the QIAquick PCR Purification Kit (Qiagen) and the quantity and quality of purified PCR products were checked using the Qubit fluorometer and a Qubit dsDNA HS kit (Thermo Scientific), both according to the respective manufacturers’ instructions.

**TABLE 1:** Primer sequences, target sites, and respective references.

Primers	Target genes	Sequence (5′–3′)	References
16S27F 23S30R 16S979F 16S544R 16S1029R	16S rRNA	AGAGTTTGATCCTGGCTCAG CTTCGCCTCTGTGTGCCTAGGT CGATGCAACGCGAAGAAC ATTCCGGATAACGCTTGC GCGCTCG TTGCGGGACTT	Taton et al., 2003; Rajaniemi-Wacklin et al., 2005; Hrouzek et al., 2005
HEPF HEPR	Microcystin/nodularin synthetase genes *mcyE*/*ndaF*	TTTGGGGTTAACTTTTTTGGGCATAGTC AATTCTTGAGGCTGTAAATCGGGTTT	Jungblut and Neilan, 2006
ndaF8452 ndaF8640	Nodularin synthetase gene *ndaF*	GTGATTGAATTTCTTGGTCG GGAAATTTCTATGTCTGACTCAG	Koskenniemi et al., 2007
mcyBHF03A mcyBHR04A	Microcystin synthetase gene *mcyB*	GCTTTAATCCACAAGAAGCTTTATTAGC CTGTTGCCTCCTAGTTCAAAAAATGACT	Hautala et al., 2013; mcyBHR04A modified from mcyBHR04

Sanger sequencing of the 16S rRNA gene was performed by Macrogen Inc. using the primers listed in Table 1. The sequences were trimmed with a phred score cutoff of 30, assembled in Unipro UGENE v36 (Okonechnikov et al., 2012) and are available under accessions MW491275-MW491284. Similar *Anabaena*/*Dolichospermum* sequences (0.0 *e*-value, > 90% identity) were identified using the NCBI BLAST (Altschul et al., 1990) and downloaded from the GenBank nr database. An alignment was prepared in UGENE, redundant (100% identical) sequences were removed and phylogenetic analyses were conducted in MEGAX (Kumar et al., 2018; Stecher et al., 2020). Two *Microcystis aeruginosa* strains were selected as an outgroup. Maximum Parsimony, Maximum Likelihood, and Neighbor Joining methods were used with the Tamura-Nei model (Saitou and Nei, 1987; Tamura and Nei, 1993; Nei and Kumar, 2000).

### Detection of 16S rRNA Gene and Cyanotoxin Genes in Sediment and Clonal Cultures

Cyanobacterial 16S rRNA gene, hepatotoxin biosynthesis genes *mcyE/ndaF*, and nodularin biosynthesis gene *ndaF* were amplified using DNA extracted from selected sediment layers and clonal cultures. gDNA, extracted as described in section “DNA Extraction, 16S rRNA Gene Amplification and Phylogenetic Analysis of Clonal Cultures,” from *Anabaena cylindrica* PCC 73105, and *Microcystis aeruginosa* PCC 7005 were used as negative controls; *Nodularia* sp. PCC 7804 was used as a positive control for nodularin; *Microcystis aeruginosa* PCC 7820, and *Dolichospermum flos-aquae* NIVA-CYA 267/4 were used as positive controls for microcystin. The strains were obtained from the Pasteur Culture Collection of Cyanobacteria and the Norwegian Culture Collection of Algae. Culture conditions and cyanotoxin data have been described earlier by Hautala et al. (2013).

The 16S rRNA gene PCR reactions were completed as described above in section “DNA Extraction, 16S rRNA Gene Amplification and Phylogenetic Analysis of Clonal Cultures.” The *mcyE/ndaF* PCR reactions were conducted in 20 μL volumes containing 8 μL template (DNA or molecular biology grade water for negative control), 1X Phire Reaction Buffer, 0.4 μL of Phire Hot Start II DNA Polymerase, 0.2 mM dNTP mix (all Thermo Fisher Scientific) and 0.4 μM of HEPF and HEPR primers (Table 1). Thermal cycling was carried out as follows: 98°C, 30 s followed by 30 cycles of 98°C, 5 s; 66°C, 5 s; 72°C, 20 s followed by a final 72°C, 1 min.

The *ndaF* PCR reactions were conducted as described above for *mcyE*/*ndaF* except with 0.4 μM of ndaF8452 and ndaF8640 primers (Table 1) and an annealing temperature of 58°C. Amplification success was examined using gel electrophoresis as described above in section “DNA Extraction, 16S rRNA Gene Amplification and Phylogenetic Analysis of Clonal Cultures.”

In order to delineate between potential microcystin and nodularin producers, the presence of the microcystin biosynthesis gene *mcyB* in sediment DNA samples was examined using PCR with primers mcyBHF03A and mcyBHR04A (Table 1) in the same way as described above for *mcyE*/*ndaF* and *ndaF*, with the following modifications: 2 μL template was used, the thermal cycling protocol was modified to consist of 35 cycles, and annealing was performed at 64°C. The specificity of the mcyBHR04A reverse primer, modified from a previously published primer mcyBHR04 (Hautala et al., 2013) by changing two nucleotides was assessed using gDNA from the culture collection strains listed above and found to yield the same results as reported in the original publication.

### Statistical Analysis

Statistical analysis was conducted using R 4.0.2 software (R Core Team, 2020) and RStudio (version 1.3.1093; RStudio Team, 2020). Spearman’s rank correlation was used to investigate the relationships between sediment depth, median sediment layer age, average akinete abundance, and hepatotoxin concentration. The significance level was set at *p* < 0.05.

## Results

### Sediment Chronology

**FIGURE 2:**
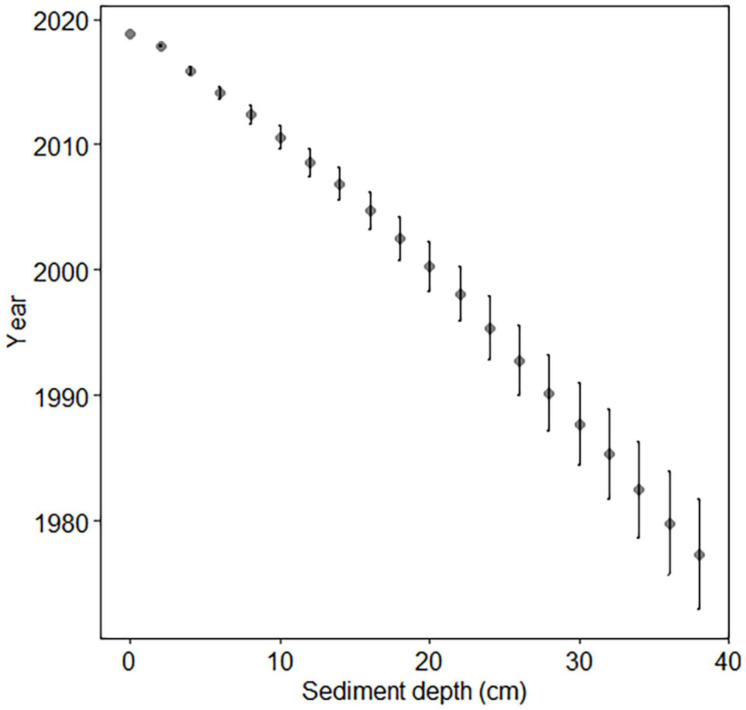
Cs-137 dating of coastal core. Error bars indicate standard deviation.

Sediment chronology was determined in order to estimate sediment and akinete age. The Cs-137 concentrations indicated that the dates of the vertical layers of the coastal sediment core ranged from late 2018 to 1977 ± 4, which corresponds with ages up to 42 ± 4 years old (Figure 2).

The open-sea site was dated previously (Kremp et al., 2018) using Pb-210 and Cs-137. Reliable age estimation extended to 16 cm depth due to the small amount of unsupported Pb-210 in the 16–18 cm region (Kremp et al., 2018). The sediment age at 16 cm depth was 105 ± 8 years, corresponding to calendar years 1914 ± 8. This is equivalent to the depth of 12 cm of the core LL7-2019 (Table 2, Supplementary Table 1, and Supplementary Figure 1), which has a slightly slower sedimentation rate (mean sedimentation rate 0.12 cm year^–1^) compared to the sedimentation rate of the core LL7-2015 (0.17 cm year^–1^; Kremp et al., 2018). Beyond the dated section, ages were estimated assuming a constant linear sedimentation rate, using a sedimentation rate of 0.05 cm year^–1^ based on the sedimentation rate of the deepest successfully dated section. Thus, it must be kept in mind that sediment ages below the depth of 12 cm (1914 AD) are only rough estimations.

**TABLE 2:** Most probable number (MPN) estimates of viable akinete abundance (akinetes g^–1^ dry weight) in three coastal and eight open-sea core sediment layers based on germination presence or absence after 4- and 8-week incubation periods.

Site	Depth (cm)	Approximate dates	Taxa	MPN 4°C		MPN 16°C	
				4 weeks	8 weeks	4 weeks	8 weeks
Coastal	2–4	2015–2017	*Anabaena/Dolichospermum* spp.	313	57,000+	57,000+	57,000+
			*Nodularia* spp.	0	6,013	0	178
	20–22	1998–2000	*Anabaena/Dolichospermum* spp.	2,270	14,917	38,000+	8,755
			*Nodularia* spp.	0	389	178	178
	38–40	1975–1977	*Anabaena/Dolichospermum* spp.	271	149	420	4,742
			*Nodularia* spp.	0	112	0	189
Open sea	2–3	2008–2011	*Anabaena/Dolichospermum* spp.	48	8,408	12,811	12,972
			*Nodularia* spp.	0	1,177	1,041	769
	8–9	1965–1971	*Anabaena/Dolichospermum* spp.	–	–	107	1,239
			*Nodularia* spp.	–	–	0	0
	10–11	1932–1952	*Anabaena/Dolichospermum* spp.	–	–	154	2,173
			*Nodularia* spp.	–	–	0	0
	12–13	1897–1916	*Anabaena/Dolichospermum* spp.	–	–	261	261
			*Nodularia* spp.	–	–	0	0
	14–15	1857–1876*	*Anabaena/Dolichospermum* spp.	–	–	354	354
			*Nodularia* spp.	–	–	0	0
	16–17	1816–1836*	*Anabaena/Dolichospermum* spp.	0	0	6.5	358
			*Nodularia* spp.	0	0	47	0
	20–21	1737–1756*	*Anabaena/Dolichospermum* spp.	0	0	0	69
			*Nodularia* spp.	0	0	0	0
	28–29	1577–1596*	*Anabaena/Dolichospermum* spp.	0	0	0	475
			*Nodularia* spp.	0	0	0	0

*The open-sea core layers 8*–*9, 10*–*11, 12*–*13, and 14*–*15 cm were only incubated at 16°C. Numbers with an indefinite MPN value (e.g., 57,000 +) indicate all dilution series replicates showed growth for that taxa. The open-sea 2*–*3 cm layer 16°C 4-week incubation MPN estimate for Aphanizomenon sp. was 5,765 akinetes g*^–^*^1^ dry weight. The dates below 13 cm (^∗^) are estimations based on a constant sedimentation rate.*

### Vertical Akinete Abundance and Cyanobacterial 16S rRNA Genes

Only intact and distinct cyanobacterial akinetes were counted to determine vertical akinete abundance. All cyanobacterial akinetes were counted, regardless of suspected species. Akinetes resembling *Nodularia* spp. were only noted in the surface layers (2–4 and 6–8 cm) of the coastal core; the rest of the akinetes found were of the *Anabaena/Dolichospermum* type (Komárek, 2013, 2016). The 2–4 cm depth was considered the surface layer of the coastal core due to the high water content (15.2 wet to dry weight ratio) of the 0–2 cm layer, which was approximately three times the average of all the layers.

**FIGURE 3:**
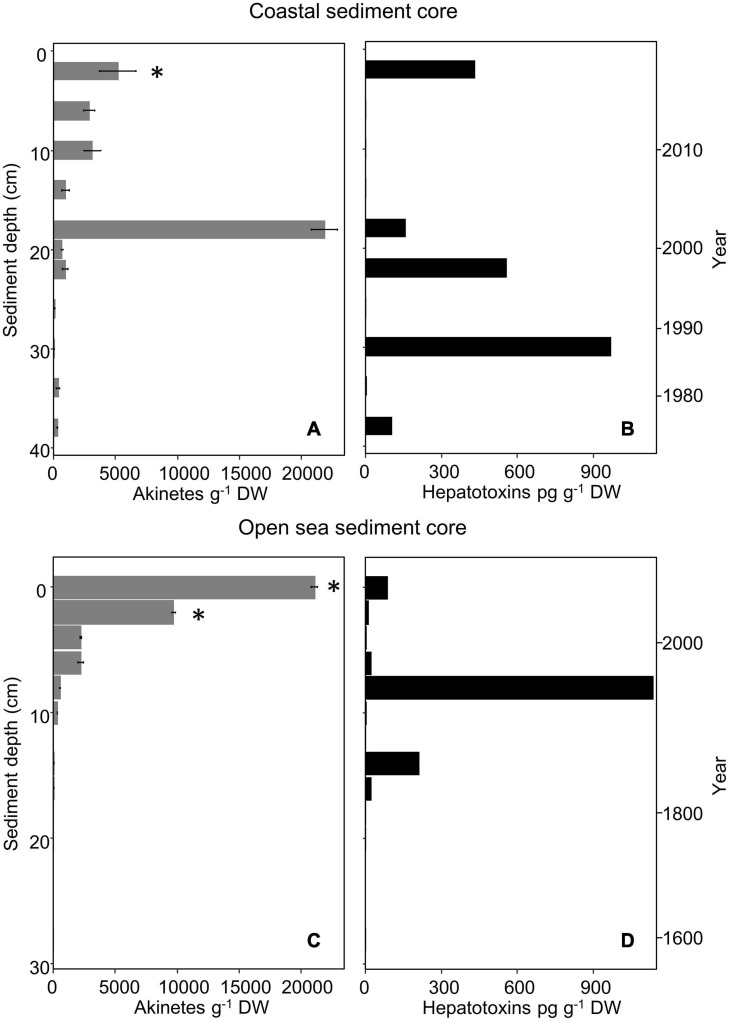
Akinete abundance (g^– 1^ dry weight; mean, *n* = 3) **(A,C)** and hepatotoxin content (pg g^– 1^ dry weight) **(B,D)** in vertical layers of the coastal **(A,B)** and open-sea **(C,D)** sediment cores. Presence of hepatotoxin genes (*mcyB, mcyE/ndaF, ndaF*) is marked with an asterisk. Error bars indicate standard deviation of akinete cell counts. Zero akinetes were observed in replicates of open-sea sediment core layers from 12–13, 20–21, and 28–29 cm depth; other depths with no value indicate that sample was not selected for analysis.

Vertical akinete abundance in the coastal sediment core ranged from approximately 22,000 to 70 akinetes g^–1^ dry weight in all examined sediment layers (Figure 3A). The highest akinete abundance (22,000 akinetes g^–1^ dry weight) was determined from the 18–20 cm layer, with an age corresponding to roughly 16–18 years. Aside from this maximum akinete abundance, the vertical abundance decreased from the surface layer abundance (5,200 akinetes g^–1^ dry weight) with depth and age with minimal variation. The oldest intact akinetes measured from the sediment core were 42 ± 4 years old. Spearman’s correlation analysis indicated a significant negative correlation between akinete abundance and both sediment depth and age, which yielded the same value (ρ = −0.82, *p* = 0.003).

Similarly, vertical akinete abundance in the open-sea core ranged from zero to approximately 21,000 akinetes g^–1^ dry weight in all examined layers of sediment (Figure 3C). The greatest akinete abundance occurred at the surface layer (0–1 cm, 0–6 years old) and decreased with depth and age. Within the accounted for 20–63 μm sediment fraction, no akinetes were present in any replicate at 12–13, 20–21, or 28–29 cm depth. The oldest intact akinetes measured directly from sediment in the open-sea core were from the depth of 18–19 cm, i.e., up to 240 years old. Spearman’s correlation analysis indicated a significant negative correlation between akinete abundance and both sediment depth and age (ρ = −0.92, *p* < 0.001).

Overall, akinete abundance was maximal in the open-sea sediment core at the surface and decreased with depth and age; whereas, in the coastal sediment core, the maximal akinete abundance occurred at the mid-depth (18–20 cm) of the core, though the akinete abundance otherwise decreased with depth and age.

DNA extraction from sediment yielded >4 ng/μL with an average A_260_/A_280_ of 1.74. All sediment layers of both cores showed positive results for the presence of cyanobacterial 16S rRNA genes (Supplementary Table 2).

### Cyanotoxin Concentration and Biosynthesis Genes in Sediment

Cyanobacterial cyclic peptide hepatotoxins (total microcystins and nodularin) were present in the 20–63 μm sediment fraction from sediment layers of both cores, even the deepest sediment layers (Figures 3B,D). For the coastal sediment core, six out of 11 analyzed samples contained concentrations above the detection limit, ranging from 2.9 to 968 pg g^–1^ dry weight. The sediment sample with the highest hepatotoxin concentration was the 30–32 cm-layer, which corresponds to calendar years 1985 to 1987. For the open-sea sediment core, eight out of nine analyzed samples contained concentrations above the detection limit, ranging from 2.7 to 1,137 pg g^–1^ dry weight. The sediment sample with the highest hepatotoxin concentration was the 8–9 cm-layer, which corresponds to calendar years 1965 to 1971.

All the samples were also analyzed by the nodularin-specific assay to detect any presence of nodularin-R in the samples. Nodularin was only detected in one of the 20 sediment samples examined: the 30–32-cm coastal sediment core layer was weakly positive for the presence of nodularin (0.12 μg L^–1^ by nodularin-specific immunoassay, equivalent to 8.7 pg g^–1^ dry weight), just above the detection limit according to the assay. Hepatotoxin concentrations did not correlate significantly with sediment depth, age, or akinete abundances at either the coastal or the open sea site (*p* > 0.05 for all).

Microcystin and nodularin biosynthesis genes *mcyB* and *ndaF* were only detectable in the 2–4 cm surface layer of the coastal sediment core (Figure 3A and Supplementary Table 2). Amplification of *mcyE*/*ndaF* was also observed in this layer. The *mcyB* gene was also detected at the open sea sediment core 0–1 and 2–3 cm layers (Figure 3C and Supplementary Table 2).

### Akinete Germination and Identification of Germinated Filaments

**FIGURE 4:**
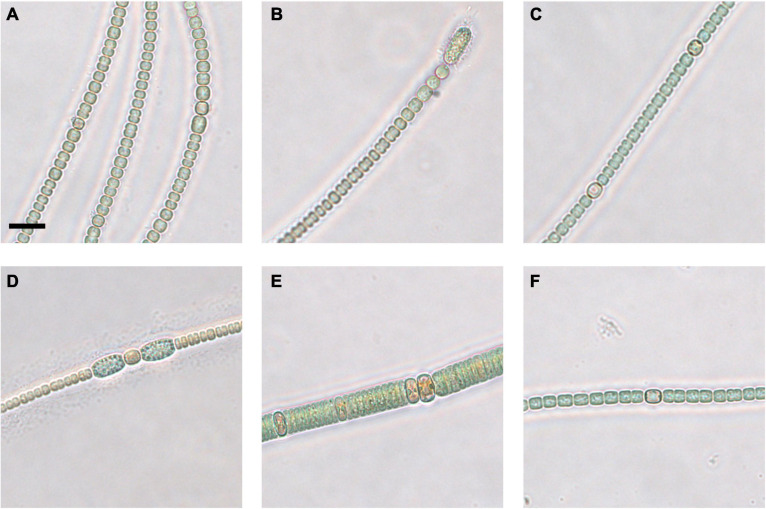
Micrographs of coastal core clonal cultured strains of *Anabaena/Dolichospermum* **(A–D,F)** and *Nodularia* **(E)**. 2–4 cm strain 1 **(A)**, strain 2 **(B)**, and strain 3 **(C)**, 20–22 cm strain 1 **(D)**, strain 2 **(E)**, and strain 3 **(F)**. Scale bar represents 10 μm.

After an 8-week incubation period at 16°C, viable akinetes had germinated from all tested sediment layers in both sediment cores (Table 2 and Figure 4). A greater number of viable akinetes germinated from the coastal sediment core than open-sea core, despite a higher akinete abundance in the upper layers of the open-sea core (Figures 3A,C). *Nodularia* spp., *Anabaena/Dolichospermum* spp., and *Aphanizomenon* sp. filaments germinated from at least one sediment layer. Germinated *Aphanizomenon* sp. filaments were only present in the 2–3 cm layer of the open-sea core in 16°C after the 4-week incubation period (MPN estimate 5,765 akinetes g^–1^ dry weight). Generally, fewer *Anabaena/Dolichospermum* spp. akinetes germinated under 4°C than 16°C conditions, excluding the four open-sea core sediment layers only incubated at 16°C.

In both the coastal and open-sea sediment cores, more *Anabaena/Dolichospermum* spp. akinetes germinated than akinetes belonging to the genus *Nodularia*. Nonetheless, the 2–4 cm coastal sediment core layer had the highest MPN viable akinete estimates for both *Anabaena/Dolichospermum* and *Nodularia* genera. Germinated *Anabaena/Dolichospermum* spp. filaments were present in samples from each examined sediment layer from both sediment cores. In the three deepest layers of the open-sea core, *Anabaena/Dolichospermum* spp. did not germinate in 4°C conditions. Germinated *Anabaena/Dolichospermum* spp. filaments were present in 16°C incubation samples even in the deepest layers of the open-sea core, dating back to the late eighteenth century. Of the coastal core sediment samples, *Nodularia* spp. filaments had generally germinated after 8 weeks of incubation. In the open-sea core, germinated *Nodularia* spp. filaments were only present in the 2–3 and 16–17 cm layers, the latter dating back to the late nineteenth century; whereas, in the coastal core, they were present in each examined layer after 8 weeks.

Germinated cyanobacteria filaments from the coastal sediment core were successfully grown in clonal culture. Nine suspected *Anabaena* or *Dolichospermum* sp. strains (Figures 4A–D,F) and one *Nodularia*-like strain (Figure 4E) were selected for Sanger sequencing and phylogenetic analysis. The *Nodularia*-like 20–22 cm strain showed a positive result for the *mcyE/ndaF* genes and a weak positive result for the *ndaF* gene. No amplification of either *mcyE/ndaF* or *ndaF* alone was observed in the other putative *Anabaena/Dolichospermum* strains.

**FIGURE 5:**
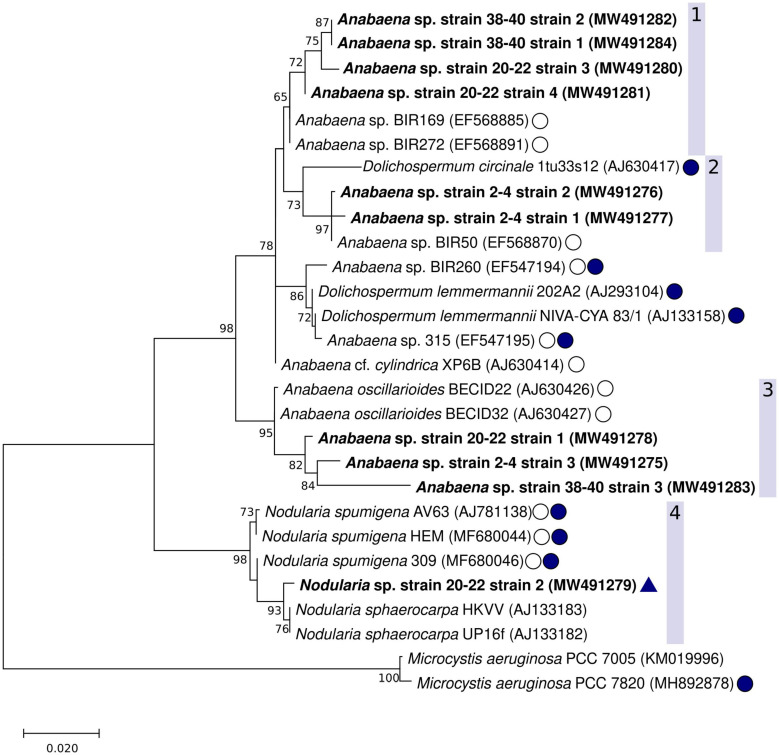
Maximum likelihood phylogenetic tree of the cyanobacterial 16S rRNA gene including the *Anabaena* sp. strains and one *Nodularia* sp. isolated in this study (shown in bold). The GenBank accession numbers are included in parenthesis with each strain. The 1196 nt alignment consisted of 28 non-redundant sequences. Bootstrap values (500 replicates) ≥ 70 are shown next to the nodes. Baltic Sea strains, other than the strains from this study, are indicated with a circle with no fill, known cyanotoxin producers (microcystins or nodularin) are indicated with filled circles. A positive result from the hepatotoxin biosynthesis gene (*mcyE*/*ndaF*) and *ndaF* PCRs (this study) is indicated with a triangle.

The Maximum Parsimony, Maximum Likelihood, and Neighbor Joining phylogenetic trees showed consistent topology, and therefore only the Maximum likelihood tree is shown (Figure 5). The cyanobacteria strains isolated from the coastal sediment core separated into distinct groups. Two well-supported clades (bootstrap values 98 and 95%, respectively) consisted of *Nodularia* spp. (Figure 5, clade 4) or benthic *Anabaena* spp. (Figure 5, clade 3). A larger clade consisting of *Anabaena* spp. such as *Anabaena* sp. strain BIR272, BIR169 and *Anabaena* cf. *cylindrica* XP6B (Halinen et al., 2008) with often similar morphology but with both benthic and planktonic lifestyles, and a planktonic *Dolichospermum* sp. strain was further divided into smaller subclades that included the remaining strains isolated in this study (Figure 5, clades 1 and 2).

Clade 1 contained suspected *Anabaena* or *Dolichospermum* sp. strains from the 20–22 and 38–40 cm-layers. The support for this cluster was moderate (65%), and the strains were found to be most closely related to one another, and then to non-toxic but planktonic *Anabaena* sp. (BIR272, BIR169). The second clade (Figure 5, clade 2) included putative *Anabaena* or *Dolichospermum* sp. strains from the sediment surface (2–4 cm-layer strains 1 and 2) as well as planktonic *Anabaena* and *Dolichospermum* strains. The strains isolated in this study were most closely related to planktonic *Anabaena* sp. BIR50, with good bootstrap support (97%). *Anabaena* sp. BIR50 has a 16S rRNA sequence identical to other Gulf of Finland strains (BIR19, BIR28, BIR30, BIR50, BIR232, BIR300, Halinen et al., 2008) which were initially identified as closely related to the sequences from this study but omitted from the analysis as redundant. *D. circinale* strain 1tu33s12, also included in clade 2, has been shown to harbor *mcy* genes (Rajaniemi et al., 2005). However, none of the strains from this study were very closely related to the planktonic, toxin-producing taxa isolated from the Baltic Sea and various lakes in Finland (Lyra et al., 2001; Rajaniemi et al., 2005).

The third clade comprised suspected *Anabaena* or *Dolichospermum* sp. strains from all three examined sediment core layers, including 2–4 cm-layer strain 3 (Figure 4C), 20–22 cm-layer strain 1 (Figure 4D), and 38–40 cm-layer strain 3, and *Anabaena oscillarioides* strains (BECID22, BECID32) isolated from epiphytic and epilithic habitats (Rajaniemi et al., 2005; Halinen et al., 2008; Shishido et al., 2015). Overall, this branch was well supported (95%).

In the fourth clade, the *Nodularia*-like 20–22 cm-layer strain 2 (Figure 4E) formed a well-supported (93%) cluster with *N. sphaerocarpa* strains (HKVV and Up16f) over other *N. spumigena* strains (309, AV63, HEM). *N. sphaerocarpa* strains are non-toxic and lack gas vesicles, though *N. sphaerocarpa* Up16f was isolated from the planktonic environment (Lehtimäki et al., 2000; Lyra et al., 2005).

## Discussion

### Vertical Akinete Abundance and Germination

This is the first study to investigate benthic archives of planktonic cyanobacterial akinetes in the Baltic Sea. Thus far, other studies hindcasting the presence of cyanobacteria in the Baltic Sea have focused only on cyanobacterial genetic and chemical markers (Bianchi et al., 2000; Funkey et al., 2014; Cegłowska et al., 2018). In this study, vertical cyanobacterial akinete abundance and germination were used to determine the persistence, viability, and species diversity of cyanobacterial akinetes in coastal and open sea regions of the northern Baltic Sea.

In the coastal sea core, the presence of cyanobacterial akinetes throughout the vertical sediment layers indicates that cyanobacteria have been continually present in the archipelago zone of the southern coast of Finland since the mid-1970s. Akinete abundance of the upper layer (2–4 cm) was roughly four times less than that of the open-sea core, which may be a result of differences in bloom magnitudes between both sites but possibly also reflect the timing of sampling. The settled late summer and early autumnal bloom was not accounted for in 2018, a significant bloom year, as coastal samples were collected well before the annual bloom started to settle. The highest akinete abundance occurred at 18–20 cm, corresponding with calendar years 2000 ± 2 to 2003 ± 2. This peak akinete abundance may represent the above average cyanobacterial blooms in the Gulf of Finland during summers of 1999 and 2002 (Kownacka et al., 2020).

In the open-sea sediment core, the presence of cyanobacterial akinetes in the depths of the core corroborates that cyanobacteria were present in open waters of the Gulf of Finland in as early as the late eighteenth century (Finni et al., 2001; Zillén and Conley, 2010; Hällfors et al., 2013). Greater akinete abundance in the upper 11 cm, dating back to the calendar year 1952, is consistent with the expansion of cyanobacterial blooms in the open Baltic Sea since the mid-twentieth century (Finni et al., 2001; Zillén and Conley, 2010; Suikkanen et al., 2013). In an investigation of Baltic Sea sediment cores, hypoxic conditions in the Baltic Sea beginning in the mid-twentieth century were linked to elevated cyanobacteria abundance (Funkey et al., 2014). The high akinete abundance (ca. 21,000 akinetes g^–1^ dry weight) of the surface layer, approximately double that of the subsequent 2–3 cm-layer, corresponds with the highest intensity bloom of the current decade in summer 2018 (Kownacka et al., 2020). At sediment depth below 11 cm a sudden drop in akinete abundance as well as MPN of viable akinetes indicates a change in deposition patterns, less intense blooms and/or reduced preservation. Generally, caution should be exercised when looking at the individual dates of the open-sea sediment core, because the dates are obtained by correlation with an earlier core from the same site. However, the dating and correlation confirm that sediment succession at the site is unmixed and the precision of the dates is adequate for the present research questions.

Under at least one of the incubation conditions for each sediment layer, the MPN viable akinete estimates exceeded akinete counts in both the open-sea and coastal sediment cores. This inconsistency is perhaps unsurprising, given that measuring akinete abundance in sediments has been cited as challenging (Pham and Utsumi, 2018; Bormans et al., 2020). This discrepancy could be the result of counting akinetes only from the 20–63 μm fraction and the MPN method bias toward culturable strains. Akinete size has not been extensively studied and tends to be quite variable, ranging from 4 to 40 μm diameter and up to ten times the size of a vegetative cell (Kaplan-Levy et al., 2010; de Tezanos Pinto et al., 2016; Sukenik et al., 2019). As such, some akinetes were likely lost in the less than 20 μm fraction, but still present in the SDC-MPN sediment slurries. Implementing an akinete-specific stain, such as SYTOX-green used by Legrand et al. (2016) or the CARD-FISH method by Ramm et al. (2012), might have helped in akinete enumeration.

Despite the inconsistency between akinete counts and MPN estimates, both values decreased with sediment age and depth in both sediment cores. This suggests that akinetes present throughout each core remained viable, but the number of akinetes and viability decreased considerably with depth. Previous studies have successfully identified akinetes from sediment samples up to 6,700 years old and germinated akinetes up to 1,830 years old from relatively shallow (<30 m) lake environments (e.g., Livingstone and Jaworski, 1980; Wood et al., 2009; Legrand et al., 2017a, 2019). Therefore, it remains plausible that akinetes from the open Gulf of Finland could be intact and viable up to >400 years old, given germination took place in even the deepest layers after 8 weeks. Only Legrand et al. (2019) has quantified akinetes and tested their ability to germinate over a 1000-year time period. Of the akinetes identified as 318 ± 40 years old in the respective study, only 6–8% germinated and were therefore considered viable, in comparison with 70% for surface sediment (Legrand et al., 2019). This decreasing rate of germination with sediment depth and age has been further corroborated by other studies and corresponds with the decrease seen in the MPN viable akinete estimates (Tsujimura and Okubo, 2003; Legrand et al., 2017b, 2019).

Various environmental factors affect cyanobacterial akinete germination, including sediment resuspension, temperature, salinity, nutrient availability, and light (Kaplan-Levy et al., 2010; Sukenik et al., 2019). The open-sea location has been reported as continually hypoxic and stratified for many decades (Raateoja and Setälä, 2016). The coastal site, on the other hand, receives a higher degree of land-based and riverine input and undergoes seasonal hypoxia and vertical mixing (Lyra et al., 2005; Gammal et al., 2017). Sediment resuspension and mixing caused by wind and wave action, such as upwelling and vertical mixing reaching up to 100 m depth, likely occurs at both sampling sites (Stipa, 1999; Suikkanen et al., 2010). Therefore, it remains feasible that akinetes from surface layers of both sediment cores could contribute to bloom initiation.

The akinete counts and MPN viable akinete estimate data indicated that akinetes play a more significant role in the life cycle of *Anabaena/Dolichospermum* spp., when compared with *Nodularia* and *Aphanizomenon* spp., as postulated in other studies (Livingstone and Jaworski, 1980; Suikkanen et al., 2010; Wasmund, 2017). However, the high MPN viable akinete estimates for *Anabaena/Dolichospermum* spp. throughout both cores are possibly due to the fact that they include both benthic and planktonic akinetes and that the MPN method favors culturable strains, such as benthic *Anabaena* spp.

*Nodularia* spp. akinetes were only observed in the upper layers (2–4 and 6–8 cm) of the coastal sediment core, which was in line with the positive hepatotoxin and *ndaF* PCR results for these layers. The negative results for the remainder of the sediment core layers, for both the coastal and open-sea sites, indicate a true absence of *N. spumigena* akinetes, as all strains are known to carry the *ndaF* gene (Laamanen et al., 2001; Moffitt et al., 2001). Based on the nodularin-specific assay all samples except for one coastal sediment core sample were negative for presence of any detectable nodularin, indicating that the hepatotoxins detected in the generic assay were predominantly microcystins. On the other hand, the very weak positive result (just above the detection limit) of the 30–32 cm coastal sediment core sample in the nodularin-specific assay does not necessarily confirm the presence of nodularin-R. The same sample was highly positive in the generic assay, indicating that most of the hepatotoxins were of microcystin origin. As the nodularin-specific assay has slight cross-reactivity with other microcystins such as microcystin-YR (Akter et al., 2017), it is also likely that the weak positive result arose from the cross-reactivity of the assay with other microcystins.

The findings of this study may corroborate the notion that *N. spumigena* overwintering strategies include a combination of the sedimentation of akinetes and trichomes in the water column (Suikkanen et al., 2010). Trichomes probably play a more significant role in bloom initiation (Wasmund, 2017), especially at open-water locations in the Gulf of Finland. This is because *N. spumigena* akinetes did not persist past the surface layer of the shallow, coastal sediment core and were absent from the deep, open-sea core. This result, however, contradicts previous research that found planktonic *N. spumigena* to germinate from sediment taken from open-sea locations and benthic *Nodularia* spp. to germinate from coastal locations (Suikkanen et al., 2010).

*Aphanizomenon* sp. was the least abundant genus among germinated filaments, present only in the upper layer (2–3 cm) of the open-sea sediment core. When compared with the biovolume of *N. spumigena* and *Dolichospermum* spp., *Aphanizomenon* sp. had the greatest average biovolume at two HELCOM monitoring stations, including the open-sea site station LL7, in the open Gulf of Finland during the summers between 1979 and 2016 (Olofsson et al., 2020). Therefore, due to high density, it remains plausible that some filaments were exported and then persisted in the bottom sediment of the open-sea site. In comparison, the absence of germinated *Aphanizomenon* sp. filaments from the coastal sediment core is surprising given the waters are less saline—and therefore favor *Aphanizomenon* sp. growth—and that blooms have been detected also in the studied coastal area previously (Laamanen et al., 2001). It is possible that the MPN values underestimated the true abundance of *Aphanizomenon* sp. akinetes because their germination was not favorable under the culture conditions. Though, it seems equally probable that Baltic Sea *Aphanizomenon* sp. do not overwinter in the form of akinetes, because previous research has found that they overwinter as vegetative filaments in the water column and are present in the water column year-round (Laamanen et al., 2002; Palińska and Surosz, 2008; Suikkanen et al., 2010; Wasmund, 2017).

### Qualitative Cyanotoxin Gene PCR, Cyanotoxin Concentration, and Phylogeny of Germinated Culturable Cyanobacteria

Hepatotoxin biosynthetic gene detection from sediment samples and germinated cyanobacterial cultures was conducted to determine the potential hepatotoxin production of vegetative strains and akinetes. The aim of this qualitative hepatotoxin gene examination in conjunction with phylogenetic analysis of culturable cyanobacteria strains was to further investigate species-specific life cycle strategies by associating germinated strains with known planktonic, benthic, or epiphytic Baltic Sea strains. The presence of cyanobacterial 16S rRNA genes throughout both sediment cores further confirms the sedimentation of cyanobacteria in both the coastal and open-sea regions of the northern Baltic Sea for the past >40 and >200 years, for coastal and open-sea sites, respectively. It is important to note, however, that the presence of the 16S rRNA gene does not necessarily indicate the sedimentation of cyanobacterial akinetes due to the presence of picocyanobacteria in the Baltic Sea.

The detection of microcystin and nodularin biosynthetic genes was limited to the surface sediment layers of both sediment cores, and therefore, the highest abundance of akinetes with hepatotoxic potential likely existed in these layers. Otherwise, however, the majority of sedimentary DNA was likely from cyanobacteria strains that did not contain microcystin or nodularin biosynthesis genes and therefore did not produce hepatotoxic blooms. Similar studies found cyanobacterial microcystin (*mcyA, mcyB, mcyD, mcyE*), nodularin (*ndaF*), and anatoxin (*anaC, anaF*) coding genes in up to 1000-years-old lake and river sediment samples using classic PCR, nested PCR, and qPCR methods (Legrand et al., 2016, 2017a, 2019; Monchamp et al., 2016; Magonono et al., 2018; Pilon et al., 2019). Consequently, it could also be useful to adapt a nested or qPCR method for lowering the detection limit of cyanotoxin genes.

Total microcystin/nodularin measurements from the 20–63 μm sediment fraction did not consistently correspond with akinete abundance or hepatotoxin gene detection. This measurement was likely affected by the timing of the analysis, which took place over a year after sediment core collection, as hepatotoxins have short half-lives in brackish sediment and are subject to microbial degradation (Kankaanpää et al., 2009). Thus, the microcystin/nodularin measurements presented here possibly reflect intracellular akinete concentrations, which is largely unknown on a per cell basis.

The immunoassay method itself is unlikely to have suffered from interference with organic materials since the 20–63 μm sediment fraction was used, rather than the raw sediment sample. Sample processing included several washing steps, which removed much of the water-soluble organic matter. Furthermore, the non-competitive immunoassay applied here is based on two-site specific recognition of microcystins/nodularin and the assay is less prone to the matrix interference compared to the competitive assay.

Culturable cyanobacteria strains germinated from the coastal core grouped together primarily with other Baltic Sea strains, including planktonic *Anabaena/Dolichospermum* spp., benthic and epiphytic *Anabaena* spp., *N. spumigena*, and *N. sphaerocarpa*. Of all 10 cultured strains, the 2–4 cm-layer strains 1 and 2 formed a group with exclusively planktonic *Anabaena* and *Dolichospermum* species, suggesting that a greater number of benthic or epiphytic cyanobacteria deposit akinetes at the coastal location. It is also possible that revived planktonic akinetes were not represented in the selected strains or in fact were less resistant to microbial degradation, as they did not persist in the sediment beyond the surface layer in this study. Further research needs to be conducted to confirm the presence of gas vacuole gene clusters in planktonic *Anabaena* spp. strains to corroborate their planktonic habitat and taxonomic classification to *Dolichospermum* (Wacklin et al., 2009; Komárek, 2010).

The mixing of benthic and planktonic strains in the second clade indicates that germinated strains could correspond with either habitat. This finding also agrees with the grouping of cyanobacteria in accordance with their morphology over their ecological niche (Lyra et al., 2005; Rajaniemi et al., 2005; Halinen et al., 2008). The third cluster of *Anabaena/Dolichospermum* spp. included strains from all three sediment layers. The monophyly, despite a 40-year age difference, could indicate a high degree of seasonal akinete recruitment from the sediment at this location among benthic strains.

Of all the cultured strains, only the 20–22 cm-layer *Nodularia*-like strain showed the potential for nodularin production. Therefore, the 20–22 cm-layer *Nodularia*-like strain may in fact be hepatotoxic *N. spumigena*, despite being more closely related to *N. sphaerocarpa* strains than *N. spumigena* included in the tree. This finding is consistent with clustering among Baltic *N. spumigena* and *N. sphaerocarpa* strains, hypothesized to be the result of similar akinete shape and size (Lyra et al., 2005; Rajaniemi et al., 2005). The detection of potential cyanotoxin production in the clonal culture but not the 20–22 cm sediment layer further supports that other PCR methods, such as nested PCR or qPCR, could allow for lowering the detection limit of cyanotoxin genes in the sediment.

Ultimately, the phylogenetic analysis indicated that the majority of culturable cyanobacteria strains from the coastal sediment core likely belonged to the benthic genus *Anabaena*. Two strains from the upper sediment layer (2–4 cm) may be *Dolichospermum* spp. because they grouped with known planktonic *Anabaena* and *Dolichospermum* taxa. Only the *Nodularia*-like strain showed positive results for hepatotoxin biosynthetic genes and grouped most closely with a Baltic Sea strain of *N. spumigena*. For many strains, phylogenetic grouping possibly resulted from similar morphology and bloom season, rather than ecological niche.

## Summary and Conclusion

In this study, cyanobacterial akinetes persisted in and germinated from northern Baltic Sea sediment up to >40 and >400 years old. Akinete abundance and viability decreased with age and depth of vertical sediment layers. Increases in akinete abundance largely corresponded with the historical expansion of anthropogenic eutrophication-fueled blooms of cyanobacteria in the northern Baltic Sea, beginning in the mid-twentieth century. The detection of potential microcystin or nodularin production from akinetes was minimal and restricted to surface sediment layers. Phylogenetic analysis of culturable cyanobacteria from the coastal sediment core indicated that most strains likely belonged to benthic species of *Anabaena*. Of the culturable cyanobacteria strains, suspected planktonic species of *Dolichospermum* only germinated from near-surface sediment layers, with an estimated age of 1–3 years. Findings also supported the notion that, in comparison with *Nodularia* and *Aphanizomenon* spp. akinetes, *Anabaena/Dolichospermum* spp. akinetes play a more significant role in their life cycle and bloom initiation strategies. Overall, there was minimal congruence between akinete abundance, cyanotoxin concentration, and the presence of cyanotoxin biosynthetic genes in either sediment core. Further research is recommended to accurately quantify all akinetes and lower the detection limit of cyanotoxin genes from brackish water sediment samples in order to further describe species-specific benthic archives of cyanobacteria.

This is the first study to research benthic archives of cyanobacterial akinetes in Baltic Sea sediment cores. Measuring cyanobacterial akinete abundance, germination experiments, and genetic methods can be effectively used to determine akinete persistence, viability, and potential cyanotoxin production in brackish water sediment samples. This study highlights the prolonged survival of cyanobacterial akinetes in northern Baltic Sea sediment. Though the present study demonstrates the higher likelihood of reviving benthic cyanobacterial species, further research should be done to confirm that Baltic Sea sediment cores can be used as a proxy to hindcast blooms of planktonic cyanobacteria. Contrasting viable akinete estimates found in this study for species of bloom-forming genera *Anabaena/Dolichospermum, Nodularia*, and *Aphanizomenon* corroborate that akinetes do not play an equally significant role in the life cycles of all nostocalean cyanobacteria in the northern Baltic Sea.

## Data Availability Statement

The datasets presented in this study can be found in online repositories. The names of the repository/repositories and accession number(s) can be found below: https://www.ncbi.nlm.nih.gov/genbank/, MW491275-MW491284.

## Author Contributions

SSu and AK conceived the study. SSu, AK, and HS designed the research. AK and SW organized the fieldwork and performed the sampling. SW, HS, and SA performed molecular and experimental work. SW, V-PV, SA, and SSa analyzed the data and SSu and HS helped with data interpretation. SW wrote the manuscript. All authors discussed the results and commented on the manuscript.

## Conflict of Interest

The authors declare that the research was conducted in the absence of any commercial or financial relationships that could be construed as a potential conflict of interest.

## References

[B1] Adams D. G. Duggan P. S. (1999). Tansley Review No. 107. Heterocyst and akinete differentiation in cyanobacteria. *New Phytol.* 144 3–33. 10.1046/j.1469-8137.1999.00505.x

[B2] Akter S. Vehniäinen M. Kankaanpää H. T. Lamminmäki U. (2017). Rapid and highly sensitive non-competitive immunoassay for specific detection of nodularin. *Microorganisms* 5:58. 10.3390/microorganisms5030058 28895936 PMC5620649

[B3] Akter S. Vehniäinen M. Spoof L. Nybom S. Meriluoto J. Lamminmäki U. (2016). Broad-spectrum noncompetitive immunocomplex immunoassay for cyanobacterial peptide hepatotoxins (microcystins and nodularins). *Anal. Chem.* 88 10080–10087. 10.1021/acs.analchem.6b02470 27657987

[B4] Altschul S. F. Gish W. Miller W. Myers E. W. Lipman D. J. (1990). Basic local alignment search tool. *J. Mol. Biol.* 215 403–410. 10.1016/S0022-2836(05)80360-2 2231712

[B5] Baker P. D. Bellifemine D. (2000). Environmental influences on akinete germination of *Anabaena circinalis* and implications for management of cyanobacterial blooms. *Hydrobiologia* 427 65–73. 10.1023/A:1003988426561

[B6] Bianchi T. S. Engelhaupt E. Westman P. Andrén T. Rolff C. Elmgren R. (2000). Cyanobacterial blooms in the Baltic Sea: Natural or human-induced? *Limnol. Oceanogr.* 45 716–726. 10.4319/lo.2000.45.3.0716

[B7] Bormans M. Savar V. Legrand B. Mineaud E. Robert E. Lance E. (2020). Cyanobacteria and cyanotoxins in estuarine water and sediment. *Aquat. Ecol.* 54 625–640. 10.1007/s10452-020-09764-y

[B8] Burford M. A. Carey C. C. Hamilton D. P. Huisman J. Paerl H. W. Wood S. A. (2019). Perspective: Advancing the research agenda for improving understanding of cyanobacteria in a future of global change. *Harmful Algae* 91:101601. 10.1016/j.hal.2019.04.004 32057347

[B9] Cegłowska M. Toruńska-Sitarz A. Kowalewska G. Mazur-Marzec H. (2018). Specific chemical and genetic markers revealed a thousands-year presence of toxic *Nodularia spumigena* in the Baltic Sea. *Mar. Drugs* 16:116. 10.3390/md16040116 29617355 PMC5923403

[B10] Ellegaard M. Clokie M. R. J. Czypionka T. Frisch D. Godhe A. Kremp A. (2020). Dead or alive: sediment DNA archives as tools for tracking aquatic evolution and adaptation. *Commun. Biol.* 3:169. 10.1038/s42003-020-0899-z 32265485 PMC7138834

[B11] Finni T. Kononen K. Olsonen R. Wallström K. (2001). The history of cyanobacterial blooms in the Baltic Sea. *Ambio* 30 172–178. 10.1579/0044-7447-30.4.172 11697246

[B12] Funkey C. P. Conley D. J. Reuss N. S. Humborg C. Jilbert T. Slomp C. P. (2014). Hypoxia sustains cyanobacteria blooms in the Baltic Sea. *Environ. Sci. Technol.* 48 2598–2602. 10.1021/es404395a 24512281 PMC3950887

[B13] Gammal J. Norkko J. Pilditch C. A. Norkko A. (2017). Coastal hypoxia and the importance of benthic macrofauna communities for ecosystem functioning. *Estuaries Coasts* 40 457–468. 10.1007/s12237-016-0152-7

[B14] Halinen K. Fewer D. P. Lyra C. Eronen E. Sivonen K. (2008). Genetic diversity in strains of the genus *Anabaena* isolated from planktonic and benthic habitats of the Gulf of Finland (Baltic Sea). *FEMS Microbiol. Ecol.* 64 199–208. 10.1111/j.1574-6941.2008.00461.x 18336556

[B15] Halinen K. Jokela J. Fewer D. P. Wahlsten M. Sivonen K. (2007). Direct evidence for production of microcystins by *Anabaena* strains from the Baltic Sea. *Appl. Environ. Microbiol.* 73 6543–6550. 10.1128/AEM.01377-07 17766456 PMC2075070

[B16] Hällfors H. Backer H. Leppänen J.-M. Hällfors S. Hällfors G. Kuosa H. (2013). The northern Baltic Sea phytoplankton communities in 1903–1911 and 1993–2005: a comparison of historical and modern species data. *Hydrobiologia* 707 109–133. 10.1007/s10750-012-1414-4

[B17] Hautala H. Lamminmäki U. Spoof L. Nybom S. Meriluoto J. Vehniäinen M. (2013). Quantitative PCR detection and improved sample preparation of microcystin-producing *Anabaena, Microcystis* and *Planktothrix*. *Ecotox*. *Environ. Safe.* 87 49–56. 10.1016/j.ecoenv.2012.10.008 23122919

[B18] Hense I. Beckmann A. (2006). Towards a model of cyanobacteria life cycle—effects of growing and resting stages on bloom formation of N2-fixing species. *Ecol. Model.* 195 205–218. 10.1016/j.ecolmodel.2005.11.018

[B19] Hinners J. Kremp A. Hense I. (2017). Evolution in temperature-dependent phytoplankton traits revealed from a sediment archive: do reaction norms tell the whole story? *Proc. R. Soc. B.* 284:20171888. 10.1098/rspb.2017.1888 29021182 PMC5647313

[B20] Hori K. Okamoto J. Tanji Y. Unno H. (2003). Formation, sedimentation and germination properties of *Anabaena* akinetes. *Biochem. Eng. J.* 14 67–73. 10.1016/S1369-703X(02)00136-5

[B21] Hrouzek P. Ventura S. Lukešová A. Mugnai M. Angela Turicchia S. Komárek J. (2005). Diversity of soil *Nostoc* strains: phylogenetic and phenotypic variability. *Algol. Stud. Suppl.* 117 251–264. 10.1127/1864-1318/2005/0117-0251

[B22] Huber A. L. (1985). Factors affecting the germination of akinetes of *Nodularia spumigena* (Cyanobacteriaceae). *Appl. Environ. Microbiol.* 49 73–78. 16346710 10.1128/aem.49.1.73-78.1985 PMC238347

[B23] Jones S. E. Lennon J. T. (2010). Dormancy contributes to the maintenance of microbial diversity. *Proc. Natl. Acad. Sci. U. S. A.* 107 5881–5886. 10.1073/pnas.0912765107 20231463 PMC2851880

[B24] Jungblut A.-D. Neilan B. A. (2006). Molecular identification and evolution of the cyclic peptide hepatotoxins, microcystin and nodularin, synthetase genes in three orders of cyanobacteria. *Arch. Microbiol.* 185 107–114. 10.1007/s00203-005-0073-5 16402223

[B25] Kankaanpää H. T. Sipiä V. O. Kuparinen J. S. Ott J. L. Carmichael W. W. (2001). Nodularin analyses and toxicity of a *Nodularia spumigena* (Nostocales, Cyanobacteria) water-bloom in the western Gulf of Finland, Baltic Sea, in August 1999. *Phycologia* 40 268–274. 10.2216/i0031-8884-40-3-268.1

[B26] Kankaanpää H. T. Sjövall O. Huttunen M. Olin M. Karlsson K. Hyvärinen K. (2009). Production and sedimentation of peptide toxins nodularin-R and microcystin-LR in the northern Baltic Sea. *Environ. Pollut.* 157 1301–1309. 10.1016/j.envpol.2008.11.044 19117649

[B27] Kaplan-Levy R. N. Hadas O. Summers M. L. Rücker J. Sukenik A. (2010). “Akinetes: Dormant cells of cyanobacteria,” in *Dormancy and Resistance in Harsh Environments*, eds Lubzens E. Cerda J. Clark M. (Berlin: Springer), 5–27. 10.1007/978-3-642-12422-8_2

[B28] Karjalainen M. Engström-Öst J. Korpinen S. Peltonen H. Pääkkönen J.-P. Rönkkönen S. (2007). Ecosystem consequences of cyanobacteria in the northern Baltic Sea. *Ambio* 36 195–202. 10.1579/0044-7447200736 17520934

[B29] Karlsson K. M. Kankaanpää H. Huttunen M. Meriluoto J. (2005). First observation of microcystin-LR in pelagic cyanobacterial blooms in the northern Baltic Sea. *Harmful Algae* 4 163–166. 10.1016/j.hal.2004.02.002

[B30] Komárek J. (2010). Modern taxonomic revision of planktic nostocacean cyanobacteria: a short review of genera. *Hydrobiologia* 639 231–243. 10.1007/s10750-009-0030-4

[B31] Komárek J. (2013). “Cyanoprokaryota. 3. Heterocytous genera,” in *Süsswasserflora von Mitteleuropa 19/3*, eds Büdel B. Gärtner G. Krienitz L. Schagerl M. (Berlin: Springer Spektrum), 1–1130.

[B32] Komárek J. (2016). Review of the cyanobacterial genera implying planktic species after recent taxonomic revisions according to polyphasic methods: state as of 2014. *Hydrobiologia* 764 259–270. 10.1007/s10750-015-2242-0

[B33] Koskenniemi K. Lyra C. Rajaniemi-Wacklin P. Jokela J. Sivonen K. (2007). Quantitative real-time PCR detection of toxic *Nodularia cyanobacteria* in the Baltic Sea. *Appl. Environ. Microbiol.* 73 2173–2179. 10.1128/AEM.02746-06 17277219 PMC1855639

[B34] Kotai J. (1972). *Instructions for preparation of modified nutrient solution Z8 for algae.* Norway: Norwegian Institute for Water Research.

[B35] Kownacka J. Busch S. Göbel J. Gromisz S. Hällfors H. Höglander H. (2020). *Cyanobacteria.* Available online at: https://helcom.fi/baltic-sea-trends/environment-fact-sheets/eutrophication/cyanobacteria-biomass/ [accessed on April 15, 2020].

[B36] Kremp A. Hinners J. Klais R. Leppänen A.-P. Kallio A. (2018). Patterns of vertical cyst distribution and survival in 100-year-old sediment archives of three spring dinoflagellate species from the northern Baltic Sea. *Eur. J. Phycol.* 53 135–145. 10.1080/09670262.2017.1386330

[B37] Kremp A. Oja J. Le Tortorec A. Hakanen P. Tahvanainen P. Tuimala J. (2016). Diverse seed banks favour adaptation of microalgal populations to future climate conditions. *Environ. Microbiol.* 18 679–691. 10.1111/1462-2920.13070 26913820

[B38] Kumar S. Stecher G. Li M. Knyaz C. Tamura K. (2018). MEGA X: Molecular evolutionary genetics analysis across computing platforms. *Mol. Biol. Evol.* 35 1547–1549. 10.1093/molbev/msy096 29722887 PMC5967553

[B39] Laamanen M. J. Forsström L. Sivonen K. (2002). Diversity of *Aphanizomenon flos-aquae* (cyanobacterium) populations along a Baltic Sea salinity gradient. *Appl. Environ. Microbiol.* 68 5296–5303. 10.1128/AEM.68.11.5296-5303.2002 12406717 PMC129895

[B40] Laamanen M. J. Gugger M. F. Lehtimäki J. M. Haukka K. Sivonen K. (2001). Diversity of toxic and nontoxic *Nodularia* isolates (Cyanobacteria) and filaments from the Baltic Sea. *Appl. Environ. Microbiol.* 67 4638–4647. 10.1128/AEM.67.10.4638-4647.2001 11571167 PMC93214

[B41] Legrand B. Lamarque A. Sabart M. Latour D. (2016). Characterization of akinetes from cyanobacterial strains and lake sediment: A study of their resistance and toxic potential. *Harmful Algae* 59 42–50. 10.1016/j.hal.2016.09.003 28073505

[B42] Legrand B. Lamarque A. Sabart M. Latour D. (2017a). Benthic archives reveal recurrence and dominance of toxigenic cyanobacteria in a eutrophic lake over the last 220 years. *Toxins* 9:271. 10.3390/toxins9090271 28869578 PMC5618204

[B43] Legrand B. Le Jeune A.-H. Colombet J. Thouvenot A. Latour D. (2017b). Akinetes may be representative of past nostocalean blooms: a case study of their benthic spatiotemporal distribution and potential for germination in a eutrophic lake. *Appl. Environ. Microbiol.* 83 1571–1517 e15. 10.1128/AEM.01571-17 28970224 PMC5691427

[B44] Legrand B. Miras Y. Beauger A. Dussauze M. Latour D. (2019). Akinetes and ancient DNA reveal toxic cyanobacterial recurrences and their potential for resurrection in a 6700-year-old core from a eutrophic lake. *Sci. Total Environ.* 687 1369–1380. 10.1016/j.scitotenv.2019.07.100 31412470

[B45] Lehtimäki J. Lyra C. Suomalainen S. Sundman P. Rouhiainen L. Paulin L. (2000). Characterization of *Nodularia* strains, cyanobacteria from brackish waters, by genotypic and phenotypic methods. *Int. J. Syst. Evol. Microbiol.* 50 1043–1053. 10.1099/00207713-50-3-1043 10843044

[B46] Livingstone D. Jaworski G. H. M. (1980). The viability of akinetes of blue-green algae recovered from the sediments of Rostherne Mere. *Br. Phycol. J.* 15 357–364. 10.1080/00071618000650361

[B47] Lyra C. Laamanen M. Lehtimäki J. M. Surakka A. Sivonen K. (2005). Benthic cyanobacteria of the genus *Nodularia* are non-toxic, without gas vacuoles, able to glide and genetically more diverse than planktonic *Nodularia*. *Int. J. Syst. Evol. Microbiol.* 55 555–568. 10.1099/ijs.0.63288-0 15774625

[B48] Lyra C. Suomalainen S. Gugger M. Vezie C. Sundman P. Paulin L. (2001). Molecular characterization of planktic cyanobacteria of *Anabaena, Aphanizomenon, Microcystis* and *Planktothrix* genera. *Int. J. Syst. Evol. Microbiol.* 51 513–526. 10.1099/00207713-51-2-513 11321098

[B49] Magonono M. Oberholster P. J. Shonhai A. Makumire S. Gumbo J. R. (2018). The presence of toxic and non-toxic cyanobacteria in the sediments of the Limpopo River Basin: Implications for human health. *Toxins* 10:269. 10.3390/toxins10070269 29970791 PMC6071004

[B50] Moffitt M. C. Blackburn S. I. Neilan B. A. (2001). rRNA sequences reflect the ecophysiology and define the toxic cyanobacteria of the genus *Nodularia*. *Int. J. Syst. Evol. Microbiol.* 51 505–512. 10.1099/00207713-51-2-505 11321097

[B51] Monchamp M.-E. Walser J.-C. Pomati F. Spaak P. (2016). Sedimentary DNA reveals cyanobacterial community diversity over 200 years in two perialpine lakes. *Appl. Environ. Microbiol.* 82 6472–6482. 10.1128/AEM.02174-16 27565621 PMC5066364

[B52] Myers J. H. Beardall J. Allinson G. Salzman S. Gunthorpe L. (2010). Environmental influences on akinete germination and development in *Nodularia spumigena* (Cyanobacteriaceae), isolated from the Gippsland Lakes, Victoria, Australia. *Hydrobiologia* 649 239–247. 10.1007/s10750-010-0252-5

[B53] Nei M. Kumar S. (2000). *Molecular Evolution and Phylogenetics.* New York: Oxford University Press.

[B54] Okonechnikov K. Golosova O. Fursov M. (2012). Unipro UGENE: a unified bioinformatics toolkit. *Bioinformatics* 28 1166–1167. 10.1093/bioinformatics/bts091 22368248

[B55] Olofsson M. Suikkanen S. Kobos J. Wasmund N. Karlson B. (2020). Basin-specific changes in filamentous cyanobacteria community composition across four decades in the Baltic Sea. *Harmful Algae* 91:101685. 10.1016/j.hal.2019.101685 32057344

[B56] Palińska K. A. Surosz W. (2008). Population of *Aphanizomenon* from the Gulf of Gdańsk (Southern Baltic Sea): differences in phenotypic and genotypic characteristics. *Hydrobiologia* 607 163–173. 10.1007/s10750-008-9388-y

[B57] Pham T.-L. Utsumi M. (2018). An overview of the accumulation of microcystins in aquatic ecosystems. *J. Environ. Manage.* 213 520–529. 10.1016/j.jenvman.2018.01.077 29472035

[B58] Pilon S. Zastepa A. Taranu Z. E. Gregory-Eaves I. Racine M. Blais J. M. (2019). Contrasting histories of microcystin-producing cyanobacteria in two temperate lakes as inferred from quantitative sediment DNA analyses. *Lake Reserv. Manage.* 35 102–117. 10.1080/10402381.2018.1549625

[B59] R Core Team (2020). *R: A language and environment for statistical computing.* Vienna: R Foundation for Statistical Computing.

[B60] Raateoja M. Setälä O. (2016). The Gulf of Finland assessment. *Rep. Finnish Environ. Inst.* 27 1–363.

[B61] Rajaniemi P. Hrouzek P. Kaštovská K. Willame R. Rantala A. Hoffmann L. (2005). Phylogenetic and morphological evaluation of the genera *Anabaena, Aphanizomenon, Trichormus* and *Nostoc* (Nostocales, Cyanobacteria). *Int. J. Syst. Evol. Microbiol.* 55 11–26. 10.1099/ijs.0.63276-0 15653847

[B62] Rajaniemi-Wacklin P. Rantala A. Mugnai M. A. Turicchia S. Ventura S. Komárková J. (2005). Correspondence between phylogeny and morphology of *Snowella* spp. and *Woronichinia naegeliana*, cyanobacteria commonly occurring in lakes. *J. Phycol.* 42 226–232. 10.1111/j.1529-8817.2006.00179.x

[B63] Ramm J. Lupu A. Hadas O. Ballot A. Rücker J. Wiedner C. (2012). A CARD-FISH protocol for the identification and enumeration of cyanobacterial akinetes in lake sediments. *FEMS Microbiol. Ecol.* 82 23–36. 10.1111/j.1574-6941.2012.01401.x 22537189

[B64] Räsänen J. Kauppila T. Vuorio K. (2006). Sediment and phytoplankton records of the cyanobacterial genus *Anabaena* in boreal Lake Pyhäjärvi. *Hydrobiologia* 568 455–465. 10.1007/s10750-006-0226-9

[B65] Rinta-Kanto J. M. Saxton M. A. DeBruyn J. M. Smith J. L. Marvin C. H. Krieger K. A. (2009). The diversity and distribution of toxigenic *Microcystis* spp. in present day and archived pelagic and sediment samples from Lake Erie. *Harmful Algae* 8 385–394. 10.1016/j.hal.2008.08.026

[B66] RStudio Team (2020). *RStudio: Integrated Development for R. RStudio.* Boston, MA: RStudio Team.

[B67] Saitou N. Nei M. (1987). The neighbor-joining method: a new method for reconstructing phylogenetic trees. *Mol. Biol. Evol.* 4 406–425. 10.1093/oxfordjournals.molbev.a040454 3447015

[B68] Savichtcheva O. Debroas D. Kurmayer R. Villar C. Jenny J. P. Arnaud F. (2011). Quantitative PCR enumeration of total/toxic *Planktothrix rubescens* and total cyanobacteria in preserved DNA isolated from lake sediments. *Appl. Environ. Microbiol.* 77 8744–8753. 10.1128/AEM.06106-11 21984244 PMC3233095

[B69] Shishido T. K. Jokela J. Kolehmainen C.-T. Fewer D. P. Wahlsten M. Wang H. (2015). Antifungal activity improved by coproduction of cyclodextrins and anabaenolysins in Cyanobacteria. *Proc. Natl. Acad. Sci. U. S. A.* 112 13669–13674. 10.1073/pnas.1510432112 26474830 PMC4640768

[B70] Sivonen K. Jones G. (1999). “Cyanobacterial Toxins,” in *Toxic Cyanobacteria in Water: A guide to their public health consequences, monitoring and management*, eds Chorus I. Bartram J. (London: The World Health Organization), 41–111.

[B71] Sivonen K. Kononen K. Carmichael W. W. Dahlem A. M. Rinehart K. L. Kiviranta J. (1989). Occurrence of the hepatotoxic cyanobacterium *Nodularia spumigena* in the Baltic Sea and structure of the toxin. *Appl. Environ. Microbiol.* 55 1990–1995. 2506812 10.1128/aem.55.8.1990-1995.1989 PMC202992

[B72] Stecher G. Tamura K. Kumar S. (2020). Molecular evolutionary genetics analysis (MEGA) for macOS. *Mol. Biol. Evol.* 37 1237–1239. 10.1093/molbev/msz312 31904846 PMC7086165

[B73] Stipa T. (1999). Water exchange and mixing in a semi-enclosed coastal basin (Pohja Bay). *Boreal Environ. Res.* 4 307–317.

[B74] Suikkanen S. Kaartokallio H. Hällfors S. Huttunen M. Laamanen M. (2010). Life cycle strategies of bloom-forming, filamentous cyanobacteria in the Baltic Sea. *Deep Sea Res. II* 57 199–209. 10.1016/j.dsr2.2009.09.014

[B75] Suikkanen S. Pulina S. Engström-Öst J. Lehtiniemi M. Lehtinen S. Brutemark A. (2013). Climate change and eutrophication induced shifts in northern summer plankton communities. *PLoS One* 8:e66475. 10.1371/journal.pone.0066475 23776676 PMC3680480

[B76] Sukenik A. Rücker J. Maldener I. (2019). “Chapter 4 - Dormant cells (akinetes) of filamentous cyanobacteria demonstrate a great variability in morphology, physiology, and ecological function,” in *Cyanobacteria, From Basic Science to Applications*, eds Mishra A. K. Tiwari D. N. Rai A. N. (London: Academic Press), 65–77. 10.1016/B978-0-12-814667-5.00004-0

[B77] Tamura K. Nei M. (1993). Estimation of the number of nucleotide substitutions in the control region of mitochondrial DNA in humans and chimpanzees. *Mol. Biol. Evol.* 10 512–526. 10.1093/oxfordjournals.molbev.a040023 8336541

[B78] Taton A. Grubisic S. Brambilla E. De Wit R. Wilmotte A. (2003). Cyanobacterial diversity in natural and artificial microbial mats of Lake Fryxell (McMurdo Dry Valleys, Antarctica): a morphological and molecular approach. *Appl. Environ. Microbiol.* 69 5157–5169. 10.1128/AEM.69.9.5157-5169.2003 12957897 PMC194958

[B79] de Tezanos Pinto P. Kust A. Devercelli M. Kozlíková-Zapomělová E. (2016). Morphological traits in nitrogen fixing heterocytous cyanobacteria: possible links between morphology and eco-physiology. *Hydrobiologia* 764 271–281. 10.1007/s10750-015-2516-6

[B80] Throndsen J. (1978). “The dilution-culture method,” in *Phytoplankton Manual*, ed. Sournia A. (Paris: UNESCO), 218–224.

[B81] Tsujimura S. Okubo T. (2003). Development of *Anabaena* blooms in a small reservoir with dense sediment akinete population, with special reference to temperature and irradiance. *J. Plankton Res.* 25 1059–1067. 10.1093/plankt/25.9.1059 32665766

[B82] Wacklin P. Hoffmann L. Komárek J. (2009). Nomenclatural validation of the genetically revised cyanobacterial genus *Dolichospermum* (Ralfs ex Bornet et Flahault) comb. nova. *Fottea* 9 59–64.

[B83] Wasmund N. (2017). Recruitment of bloom-forming cyanobacteria from winter/spring populations in the Baltic Sea verified by a mesocosm approach. *Boreal Environ. Res.* 22 445–455.

[B84] Wood S. A. Jentzsch K. Rueckert A. Hamilton D. P. Cary S. C. (2009). Hindcasting cyanobacterial communities in Lake Okaro with germination experiments and genetic analyses. *FEMS Microbiol. Ecol.* 67 252–260. 10.1111/j.1574-6941.2008.00630.x 19077032

[B85] Yan D. Xu H. Yang M. Lan J. Hou W. Wang F. (2019). Responses of cyanobacteria to climate and human activities at Lake Chenghai over the past 100 years. *Ecol. Indic.* 104 755–763. 10.1016/j.ecolind.2019.03.019

[B86] Zillén L. Conley D. J. (2010). Hypoxia and cyanobacteria blooms - are they really natural features of the late Holocene history of the Baltic Sea? *Biogeosciences* 7 2567–2580. 10.5194/bg-7-2567-2010

