# Supplementary material for: Cyanobacterial Akinete Distribution, Viability, and Cyanotoxin Records in Sediment Archives From the Northern Baltic Sea
Source: Front Microbiol. 2021 Jun 15;12:681881. doi: 10.3389/fmicb.2021.681881 (PMC8241101; doi:10.3389/fmicb.2021.681881)
Supplement: Supplementary file 1 [file Data_Sheet_1.docx]

Supplementary Material

# Supplementary Data 1. Detection of hepatotoxins by non-competitive immunoassay

The modified non-competitive immunoassay for microcystin/nodularin (Akter et al., 2016) was performed as follows: 25 µL of reagent water (for blank measurement), microcystin-LR standard solution (0.01 to 100 µg L^-1^, prepared in reagent water, certified microcystin-LR calibration solution obtained from the National Research Council of Canada), or diluted sediment extracts were added to prewashed streptavidin-coated wells (Kaivogen Oy, Finland). For blank measurements 16 replicate wells and for standards and samples duplicate wells were used. Reagent mixture containing all other assay components (biotinylated anti-ADDA antibody 1 µg mL^-1^ (Enzo Life Sciences Inc., USA), anti-immunocomplex ScFv-alkaline phosphatase SA51D1 1 µg mL^-1^ (University of Turku, Finland) and bacterial anti-alkaline phosphatase polyclonal antibody 0.5 µg mL^-1^ (LifeSpan Biosciences, Inc., USA) labelled with europium at the University of Turku) were added to each well as 50 µL, making the final reaction volume 75 µL. The plates were incubated for 1 h with slow shaking followed by four washes. Finally, 200 µL of Europium fluorescence intensifier solution (Kaivogen Oy, Finland) was added to each well and after a 10 min incubation as above, time-resolved fluorescence signal of europium was measured using a Victor 1420 Multilabel Counter (Wallac/PerkinElmer Life Sciences, USA). The detection limit (expressed in microcystin-LR equivalents) of the immunoassay was 0.04 µg L^-1^, based on the average + 3 SD (standard deviation) of blank measurements (16 replicates).

The nodularin-specific immunoassay protocol was similar to described above except that nodularin- R was used as standard and anti-immunocomplex ScFv-alkaline phosphatase SA32C11 (Akter et al., 2017) was used instead of SA51D1. The detection limit (expressed in nodularin-R equivalents) of the immunoassay was 0.011 µg L^-1^, based on the average + 3 SD of blank measurements (17 replicates).

**Supplementary Table 1.** Correlation of the two open sea sediment cores taken from station LL7 and the ages of the correlated depth layers in core LL7-2019. The ages are based on a CRS model by Kremp et al. (2018), available until the depth of 16 cm in the LL7-2015 core, corresponding to 12 cm in the LL7-2019 core. The ages of deeper sediment layers are based on assumption of constant linear sedimentation rate indicated with *gray and italic.*

| **LL7-2015 depth (cm)** | **LL7-2019 depth (cm)** | **Age in LL7-2019** |
| --- | --- | --- |
| 2-3 | 2-3 | 2008-2011 |
| 3-4 | 3-4 | 2003-2008 |
| 7-8 | 5-6 | 1987-1996 |
| 9-10 | 6-7 | 1978-1987 |
| 12-13 | 9-10 | 1952-1965 |
| 15-16 | 11-12 | 1916-1932 |
| 17-18 | 12-13 | *1897-1916* |
| 19-20 | 14-15 | *1857-1876* |
| 23-24 | 18-19 | *1777-1796* |
| 26-27 | 20-21 | *1737-1756* |
| - | 28-29 | *1577-1596* |

**Supplementary Table 2.** Extracted gDNA from sediment layers tested for the presence of cyanobacterial 16S rRNA, hepatotoxin genes (*mcyE/ndaF*), microcystin gene *mcyB*, and nodularin gene *ndaF*. + indicates a positive result; – indicates a negative result; (+) indicates a weak positive result.

| **Site** | **Depth (cm)** | **16S rRNA** | **Hepatotoxin (*mcyE/ndaF*)** | ***mcyB*** | ***ndaF*** |
| --- | --- | --- | --- | --- | --- |
| Coastal | 2-4 | + | + | + | (+) |
|  | 6-8 | + | – | – | – |
|  | 10-12 | + | – | – | – |
|  | 14-16 | + | – | – | – |
|  | 18-20 | + | – | – | – |
|  | 20-22 | + | – | – | – |
|  | 22-24 | + | – | – | – |
|  | 26-28 | + | – | – | – |
|  | 30-32 | + | – | – | – |
|  | 34-36 | + | – | – | – |
|  | 38-40 | + | – | – | – |
| Open sea | 0-1 | + | – | + | – |
|  | 2-3 | + | – | + | – |
|  | 4-5 | + | – | – | – |
|  | 6-7 | + | – | – | – |
|  | 8-9 | + | – | – | – |
|  | 10-11 | + | – | – | – |
|  | 12-13 | + | – | – | – |
|  | 14-15 | + | – | – | – |
|  | 16-17 | + | – | – | – |
|  | 18-19 | + | – | – | – |
|  | 20-21 | + | – | – | – |
|  | 28-29 | + | – | – | – |


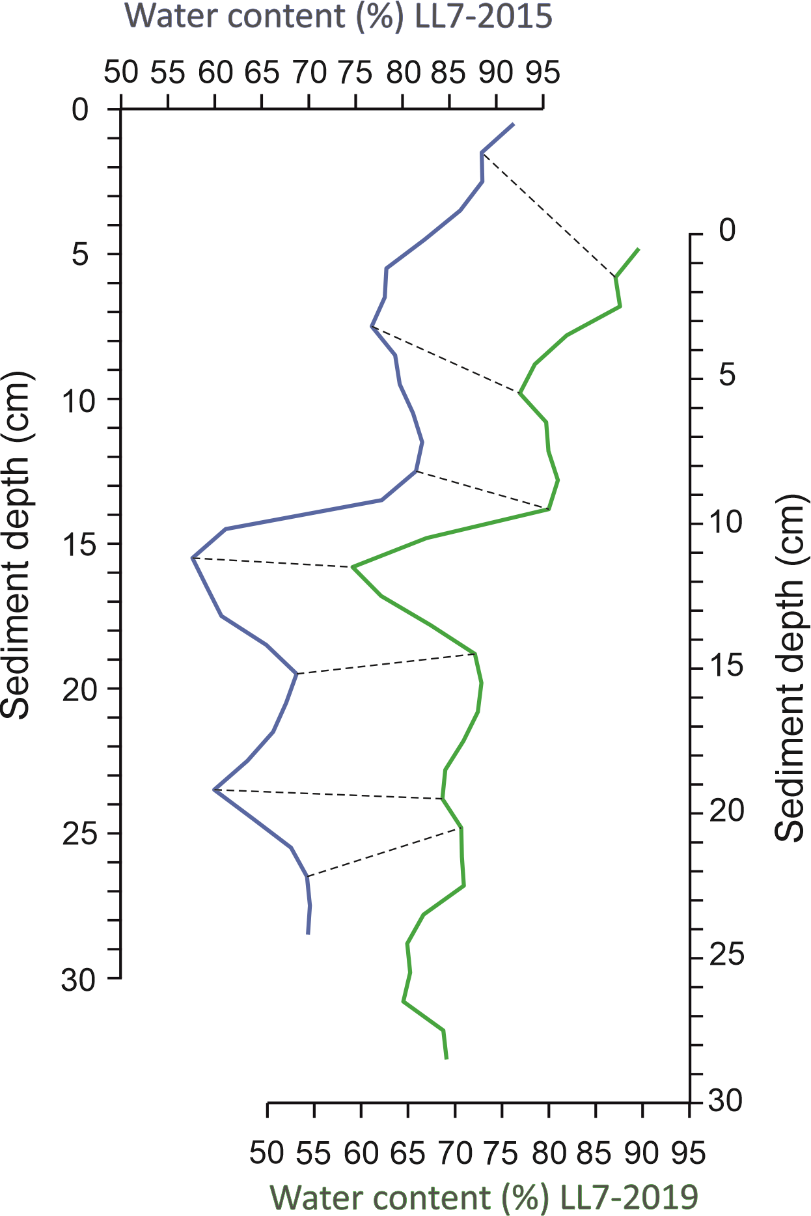


**Supplementary Figure 1.** Correlation of the two open sea sediment cores (LL7-2015 and LL7-2019) using water content changes of the sediment sequences.
